# Diffuse Calcifications Protect Carotid Plaques regardless of the Amount of Neoangiogenesis and Related Histological Complications

**DOI:** 10.1155/2015/795672

**Published:** 2015-03-25

**Authors:** Francesco Vasuri, Silvia Fittipaldi, Rodolfo Pini, Alessio Degiovanni, Raffaella Mauro, Antonia D'Errico-Grigioni, Gianluca Faggioli, Andrea Stella, Gianandrea Pasquinelli

**Affiliations:** ^1^Pathology Unit, Department of Experimental, Diagnostic and Specialty Medicine (DIMES), S. Orsola-Malpighi Hospital, Bologna University, Via Massarenti 9, 40138 Bologna, Italy; ^2^Vascular Surgery Unit, Department of Experimental, Diagnostic and Specialty Medicine, S. Orsola-Malpighi Hospital, Bologna University, Via Massarenti 9, 40138 Bologna, Italy

## Abstract

*Background.* Neoangiogenesis is crucial in plaque progression and instability. Previous data from our group showed that Nestin-positive intraplaque neovessels correlated with histological complications. The aim of the present work is to evaluate the relationship between neoangiogenesis, plaque morphology, and clinical instability of the plaque. *Materials and Methods.* Seventy-three patients (53 males and 20 females, mean age 71 years) were consecutively enrolled. Clinical data and 14 histological variables, including intraplaque hemorrhage and calcifications, were collected. Immunohistochemistry for CD34 and Nestin was performed. RT-PCR was performed to evaluate Nestin mRNA (including 5 healthy arteries as controls). *Results.* Diffusely calcified plaques (13/73) were found predominantly in females (*P* = 0.017), with a significantly lower incidence of symptoms (TIA/stroke (*P* = 0.019) than noncalcified plaques but with the same incidence of histological complications (*P* = 0.156)). Accordingly, calcified and noncalcified plaques showed similar mean densities of positivity for CD34 and Nestin. Nestin density, but not CD34, correlated with the occurrence of intraplaque hemorrhage. *Conclusions.* Plaques with massive calcifications show the same incidence of histological complications but without influencing symptomatology, especially in female patients, and regardless of the amount of neoangiogenesis. These results can be applied in a future presurgical identification of patients at major risk of developing symptoms.

## 1. Introduction

The vulnerable atheromatous plaques have been originally described as characterized by a large lipid core, a thin fibrous cap, a rich infiltrate of macrophages, and little smooth muscle cell component [1]. Neoangiogenesis began to stand out as one of the most important pathological processes involved in the plaque progression only in the recent years [2], when neovessel formation was related to an increased plaque vulnerability and to the onset of clinical symptoms [3, 4]. In particular, the intraplaque hemorrhage and the incidence of symptomatic plaques were directly related to the neovessel density, simply measured by means of immunohistochemistry (IHC) for CD34 [3]. If the density of neovessels is likely to be directly related with plaque growth and progression [5], the morphology of the neoangiogenetic structures plays a key role in the onset of the plaque instability. In fact plaque neovessels are reported to lack extracellular junctions [6]; moreover, symptomatic plaques show larger and more irregular neoangiogenetic structures compared to the neovessels of asymptomatic plaques [7]. Recently we described the IHC and immunofluorescence positivity for Nestin and WT1 in* vasa vasorum* from healthy arteries, hypothesizing that they might represent the starting point of the neoangiogenesis during atherosclerosis [8]. Afterwards we actually confirmed the expression of Nestin and WT1 in diseased arteries but together with the observation that nearly 36% of the neovessels showed positivity for Nestin and negativity for WT1 (Nestin+/WT1−). As a matter of fact, hemorrhagic plaques showed significantly more Nestin+/WT1− neoangiogenesis than uncomplicated plaques at both IHC and RT-PCR [9].

The aims of the present study were (i) to evaluate the relationship between the intraplaque neoangiogenesis (as quantified by CD34 and Nestin IHC) and the main histopathological plaque characteristics (especially complications and calcifications) and (ii) to evaluate the relationship between the immunohistochemical and histopathological characteristics and the clinical plaque instability.

## 2. Materials and Methods

### 2.1. Patients and Clinical Data

This study was carried out in conformity to the ethical guidelines of the 1975 Declaration of Helsinki (and following modifications); informed consent was obtained from all patients before surgery. We evaluated all consecutive cases of carotid endarterectomy specimens that came to our Surgery Unit in 2 years, from January 2011. According to the European Society for Vascular Surgery (ESVS) and the Society of Vascular Surgeons (SVS) recommendations [11, 12], the patients were submitted to carotid endarterectomy (CEA) for either symptomatic carotid plaques ≥50% or asymptomatic carotid stenosis ≥70%. For each patient the following clinical data were collected: occurrence of symptoms related to the carotid disease, that is, transient ischemic attack (TIA) and stroke, association with chronic ischemic cardiopathy, chronic obstructive bronchopneumonia and/or chronic renal failure, smoke, diabetes, dyslipidemia, and therapy with acetylsalicylic acid or statins.

### 2.2. Histopathological Analysis

Endarterectomy specimens were sent to our Pathology Unit, fixed in formalin, routinely processed, and paraffin embedded. Two *μ*m thick slices were cut from the paraffin blocks for haematoxylin-eosin and trichrome stains. For each case, the following histopathological features were collected as single variables, as previously reported [9]: the occurrence of intraplaque complications (hemorrhage, thrombosis, and/or surface defects) which put the plaque into the AHA type VI [10], maximum and minimum size of the fibrous cap, extension of the lipid core, and extension of the inflammatory infiltrate. For the purposes of the study, the amount of calcifications was assessed and graded from 0 to 4+ in relation to their extension along the vessel circumference; the intraplaque calcification was therefore simplified in low-grade (if 0 to 2+) and high-grade (if 3+ or 4+).

### 2.3. Immunohistochemistry

The monoclonal antibodies used in this study are listed in Table 1. Immunohistochemistry (IHC) for Nestin was performed manually, as previously described [8, 9]. IHC for CD34 was performed automatically, by means of Benchmark XT (Ventana Medical System), using the XT ultraView DAB v3 program.

The microvessel “density of positivity” [9] for Nestin was evaluated after the identification of specific Regions of Interest (ROI). ROI were defined as areas with CD34-positive neoangiogenesis, and at 20x magnification each ROI was divided into 1 mm^2^ fields using an Olympus ocular micrometer (1 length unit = 5 *μ*m, which means that an area of 100 × 100 units is equal to 0.25 mm^2^). Firstly, CD34 and Nestin microvessel positivity were counted separately; the Nestin-positive vessels were counted in the same ROI where CD34 was evaluated. Afterwards we calculated the “density of positivity” for CD34 and Nestin by dividing the sum of all the positive vascular structures observed by the number of the counted fields in each section. Finally we calculated the ratio between the densities of positivity of Nestin and CD34 in each case: the Nestin/CD34 ratio represents how many CD34-positive neovessels concomitantly express Nestin in the intraplaque neoangiogenesis.

### 2.4. RT-PCR

Reverse Transcriptase-Polymerase Chain Reaction (RT-PCR) for the product of the Nestin gene was carried out on 5 type V and on 5 type VI plaques, in order to evaluate the different expression in complicated and uncomplicated carotid plaques. A pool composed of 5 healthy carotid arteries was used as controls. Healthy carotids were kindly provided by the Cardiovascular Tissue Bank of S. Orsola-Malpighi University Hospital of Bologna from 5 multiorgan donors (3 males and 2 females, mean age 33.8 ± 13.2 years, range 18–53 years), without known comorbidities.

Tissues were homogenized with an Ultraturax and incubated with 800 ul of Trizol reagent (TRIzol Life Technologies, Carlsbad, CA, USA) for 5 min at RT. RNA extraction with Trizol was performed following manufacturer instructions. RNA quality and concentration were measured by using an ND-1000 spectrophotometer (NanoDrop, Thermo Fisher Scientific, Wilmington, DE, USA). Reverse transcription assay was performed using 2.0 *μ*g of starting total RNA quantity per 25 *μ*L of mix, following the manufacturer's protocol (High capacity cDNA Archive kit, Life Technologies). The cDNA was stored at −20°C until RT-PCR was performed. RT-PCR was carried out following MasterMix TaqMan Protocol (TaqMan Univ PCR MasterMix, Life Technologies). Four *μ*L of neat cDNA was amplified using specific probes for Nestin (NC_000001.10) and GUSB (NM_000181.3) in the RT-PCR mix (TaqMan Gene Expression Assay, Life Technologies, respective ID assay: Hs04187831_g1, Hs00939627_m1). Reactions were run on ABI PRISM 7900HT Sequence Detection System (Life Technologies). Cycling conditions were as follows: 10 min at 95°C, 50 cycles at 95°C for 15 s, and 60°C for 60 sec. Each assay was carried out in triplicate and the transcription level was normalized using GUSB as a reference gene.

### 2.5. Statistical Analysis

All statistical analyses were carried out with SPSS software for Windows, version 20. All continuous variables are expressed as means, standard deviations, and ranges; all categorical variables (both nominal and ordinal) are expressed as number of cases and percentages. The Spearman test, the chi-square test, the Mann-Whitney *U* test, and the Kruskal-Wallis test were used when appropriate. The mRNA expression values for atheromatous type V and type VI plaques are presented as fold expression in relation to healthy arteries; the actual values were calculated using the 2^−ΔΔCT^ equation, where ΔΔCT = [CT Target − CT GUSB] (atheromatous sample V or VI) − [CT Target − CT GUSB] (healthy sample). RT-PCR data were analyzed assuming the null hypothesis that the CT differences between target and reference genes will be the same in type V tissue versus type VI tissue. If the null hypothesis is not rejected, then the ΔΔCT would not be significantly different from 0. Analyses of differences between the three groups (healthy, class V, and class VI plaques) were performed with one-way ANOVA test, followed by Tukey's test. All the *P* values are derived from testing the null hypothesis that ΔΔCT are equal to 0 (at *P* = 0.05). SEM, SD, and the confidence interval (CI) of 2^−(ΔΔCT)^ are all derived from the SEM, SD, and CI of ΔΔCT as explained by Yuan et al. [13].

## 3. Results

### 3.1. Patients and Histopathological Analysis

Seventy-three patients were finally enrolled, 53 (72.6%) males and 20 (27.4%) females, with a mean age at the time of endarterectomy of 70.8 ± 8.7 years (range 42–86 years). The clinical characteristics of the patients, including ongoing therapy, are summarized in Table 2. Notably, 29 (39.7%) plaques were symptomatic, since 11 (15.1%) patients had a stroke as clinical presentation and 18 (24.6%) had a transient ischemic attack (TIA).

At histopathological analysis, mean maximum cap size was 1132.8 ± 485.6 *μ*m (range 120–2500 *μ*m) and mean minimum cap size was 284.3 ± 199.7 *μ*m (range 40–1125 *μ*m); the lipid core was not evident in 8 (11.0%) cases, 1/4 in 16 (21.9%), 2/4 in 18 (24.7%), 3/4 in 23 (31.4%), and 4/4 in 8 (11.0%); the inflammatory infiltrate was mild/focal or absent in 14 (19.2%) cases, moderate in 20 (27.4%) cases, and severe/diffuse in 39 (53.4%) cases. Fifty-two (71.2%) plaques were classified as AHA type VI [10]: the most common complication observed was intraplaque hemorrhage, present in 41 cases, followed by endothelial erosion in 22 cases and thrombosis in 4 cases. Eight (11.0%) further noncomplicated plaques were classified as type VII due to the prevalently calcified core in 8 (11.0%) cases, while the remaining 13 (17.8%) cases were classified as type VIII (prevalently fibrotic core) or type V (fibroatheroma) [10].

**Figure 1:**
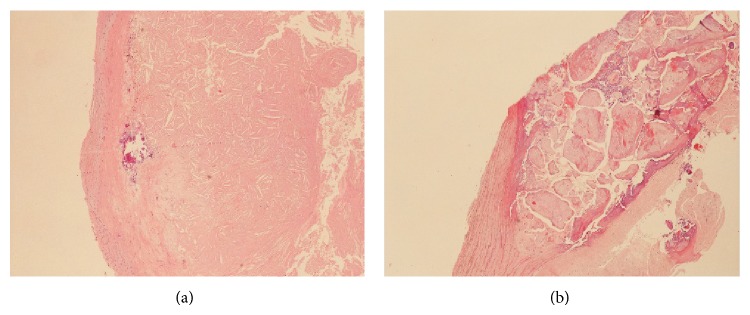
Details of two cases of carotid plaque with low-grade calcification (a) and high-grade calcification (b), respectively. Haematoxylin-eosin stain, magnification 10x.

Intraplaque calcifications were graded 0 in 11 (15.1%) cases, 1+ in 21 (28.8%), 2+ in 20 (27.4%), 3+ in 16 (21.9%), and 4+ in 5 (6.8%). According to this semiquantitative assessment of the calcification extent, low-grade calcifications (up to 2+) were recorded in 52 (71.2%) plaques, and high-grade calcification (3+ and 4+) was recorded in 21 (28.8%) plaques (Figure 1).

Finally, according to the occurrence of intraplaque complications and/or calcifications, our cases were sorted in noncalcified complicated plaques (type VI-nc, *N* = 39), calcified and complicated plaques (type VI-c *N* = 13), calcified noncomplicated plaques (type VII, *N* = 8), and noncalcified noncomplicated plaques (types V–VIII, *N* = 13). No correlations were found between plaque morphological criteria used in the present study (i.e., calcifications and histological complications) and the occurrence of chronic ischemic cardiopathy, chronic obstructive bronchopneumonia, chronic renal failure, smoke, diabetes, hypertension, dyslipidemia, or therapy with acetylsalicylic acid or statins (data not shown, chi-square test).

### 3.2. Nestin-Positive Neoangiogenesis

Two cases had no appreciable intraplaque neoangiogenesis after IHC for CD34. In the remaining 71 cases, the mean density of positivity for CD34 was 10.1 ± 3.9 structures/field (range 3.5–22.1/field). The mean density of positivity for Nestin, evaluated in the same ROI, was 6.8 ± 3.7 structures/field (range 1.4–18.5/field). The mean Nestin/CD34 ratio was 0.7 ± 0.2, which means that 70% of the CD34-positive neovessels examined coexpressed Nestin. This result is in line with what is previously described [9].

The total amount of neoangiogenesis, expressed as CD34-positive vessels, was not significantly different between type VI plaques and uncomplicated plaques (*P* = 0.111, Mann-Whitney *U* test), while the density of Nestin-positive neovessels was significantly higher in type VI (*P* = 0.015, Table 3).

**Figure 2:**
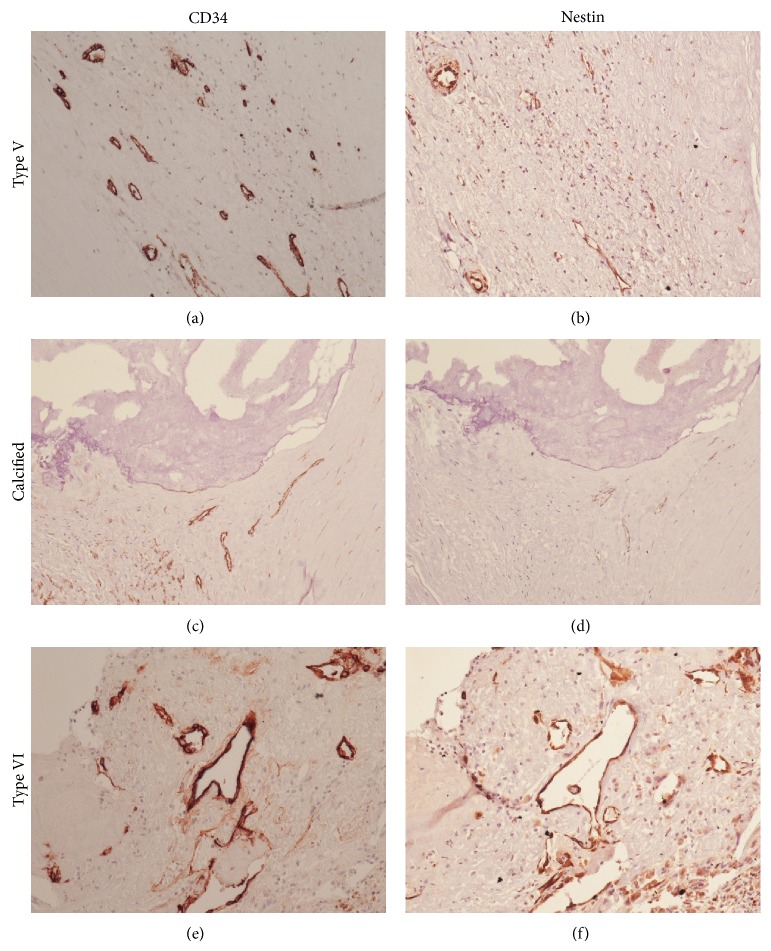
Immunohistochemical staining for CD34 (a), (c), and (e) and Nestin (b), (d), and (f) in an uncomplicated noncalcified plaque (a) and (b), a calcified plaque (c) and (d), and a complicated noncalcified plaque (e) and (f). Neoangiogenesis in uncomplicated plaques is generally Nestin-negative. The overall neoangiogenesis in calcified plaques (complicated or not) is generally lower (both CD34 and Nestin) than in complicated plaques. Magnification 20x.

The calcified plaques (including complicated and uncomplicated) showed overall less neoangiogenesis, measured with both CD34 and Nestin, than noncalcified plaques (*P* < 0.001, Table 3; Figure 2).

**Figure 3:**
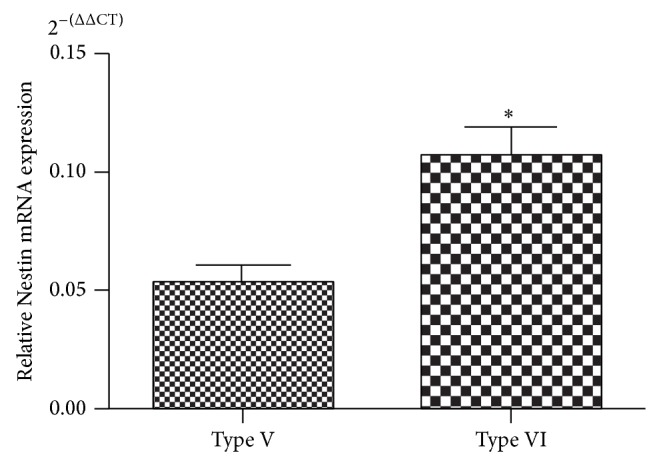
Difference in Nestin mRNA expression between type V (uncomplicated) and type VI (complicated) plaques (^*^ *P* < 0.001).

At RT-PCR, the total mean extracted mRNA from healthy tissue and type V and type VI plaques was 8764 ng, 5069.4 ng, and 2172 ng, respectively. The mean CT values of endogenous control GUSB were 36.28 ± 0.21 in healthy tissue, 31.36 ± 0.32 in type V plaques, and 34.20 ± 0.22 in type VI plaques. Mean CT for tested gene Nestin were 33.04 ± 0.06 in healthy tissue, 32.37 ± 0.12 in type V plaques, and 34.21 ± 0.30 in type VI plaques. ΔΔCT Nestin was significantly different from 0 (*P* = 0.0001); thus the null hypothesis was rejected, which indicated a change in Nestin gene expression among healthy, type V and type VI plaques. In type V and type VI plaques, the mean ΔΔCT Nestin was, respectively, 4.25 and 3.25; this corresponds to 2^−(ΔΔCT)^ of 0.05 for Nestin gene expression in type V plaques and 0.11 in type VI plaques. The type VI plaques showed a 2-fold expression increase for Nestin gene compared to type V plaques, as a confirmation of the IHC results (Figure 3).

These data confirm that Nestin-positive neoangiogenesis, studied both on the protein (IHC) and the mRNA level (RT-PCR), plays a key role in the development of intraplaque complications and that plaques with massive calcifications generally have a minor density of neoangiogenesis compared to other plaques.

### 3.3. Intraplaque Calcifications and Clinical Stability

**Figure 4:**
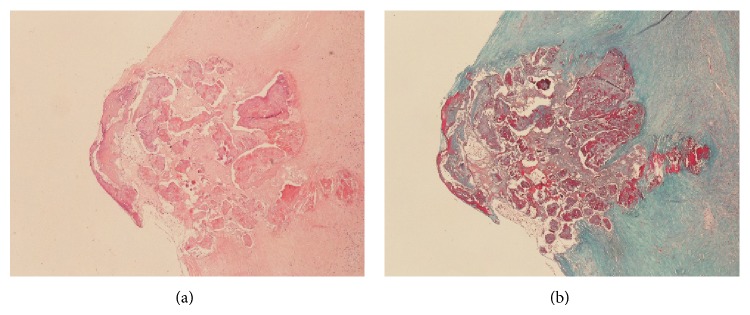
An example of calcified plaque with histopathological complications (i.e., hemorrhage) with haematoxylin-eosin stain (a). The trichrome stain highlights the hemorrhagic foci (in red (b)). Magnification 10x.

Despite the differences in neoangiogenesis, the incidence of histological complications did not differ significantly between calcified and noncalcified plaques (*P* = 0.167, chi-square test; Figure 4).

As for the clinical presentation, unsurprisingly, 25 out of 29 (86.2%) symptomatic patients (i.e., with stroke or TIA) had a type VI plaque, versus 27 out of 44 (61.4%) asymptomatic patients (*P* = 0.026, chi-square test). Interestingly, among the 21 patients with calcified plaques, only 4 (19.0%) had symptoms at the onset, regardless of the occurrence of histological complications; conversely 25 out of 52 (48.1%) patients with noncalcified plaques were symptomatic (*P* = 0.019, chi-square test).

The patients' gender was correlated with the plaque morphology and instability as well; first of all only 3 (15.0%) of the 20 female patients in our study were symptomatic versus 26 (49.1%) out of 53 males (*P* = 0.007, chi-square test). All three symptomatic female patients had a type VI-nc atheromatous lesion. Notably, a higher incidence of calcified plaques was observed in female patients (*P* = 0.017): 10 (50.0%) females had calcified plaques (5 type VII and 5 type VI-c) versus only 11 (20.8%) males (3 type VII and 8 type VI-c).

## 4. Discussion

The severity and extent of calcification reflect the atherosclerotic plaque burden and strongly predict cardiovascular morbidity and mortality [14, 15]; in a relatively recent study, no patients were found to have calcifications confined only to the coronary or carotid beds [16]. The extent of calcification is associated with a worse prognosis, albeit the real impact of calcifications within a specific vascular pathological district remains unclear [17]. For example, in the coronary vessels small calcium depositions increase the probability of atherosclerotic plaque rupture, especially on their edges, while massive calcification seems to be associated with a decreased risk [17, 18]. Anyway, vascular calcification is considered a worsening factor, probably due to its coexistence with the general risk factors; a study by Iribarren et al. [19] found that aortic arch calcification was associated with coronary heart disease risk both in men and in women. Thus aortic arch calcification may reflect the general burden of disease or be a marker of more aggressive disease. At any chance, the clinical impact of a plaque in which both calcification and histological complications coexist is far from being clarified.

Our aims were to evaluate the relationship between the intraplaque neoangiogenesis, the main histopathological characteristics (histological complications and calcifications), and the clinical plaque instability. For these purposes, neoangiogenesis was evaluated and semiquantified by CD34 and Nestin IHC, followed by RT-PCR. The majority of tissue obtained from endarterectomy was used for the routine histological diagnosis of surgical specimens. The remaining tissue had to be fully processed for RT-PCR and Western blot analysis was not included in our protocol. However, in our experience [8, 9] we observed that Nestin staining in IHC is very reliable, allowing us to evaluate its cell location and its expression at a protein level as well, without the need of the more sensible immunoblot techniques. The correlation between IHC and RT-PCR confirmed that there is a direct relationship between Nestin protein and mRNA.

According to our data calcified plaques show less inflammatory infiltrate, a smaller lipid core, and less neoangiogenesis than other plaques, but with the same incidence of hemorrhage, thrombosis, and surface defects, which define the plaque as histologically complicated [10]. Yet, interestingly, in these calcified plaques complications are not correlated with clinical plaque instability (evaluated as symptomatology); indeed, the incidence of TIA/stroke in patients with calcified plaques was sensibly lower than patients with noncalcified plaques, despite the same incidence of intraplaque complications. This is noteworthy, since at least in the carotid district the presence of calcifications seems to imply a sort of clinical “protection” to complications, making the histological complications play second fiddle. For these reasons, in our opinion, these plaques can be classified among the type VII plaques, instead of type VI, at least on clinical grounds. Alternatively, they can be classified as type VI, but the extension of the calcifications should be stated in the pathological report, to highlight their protective nature.

**Figure 5:**
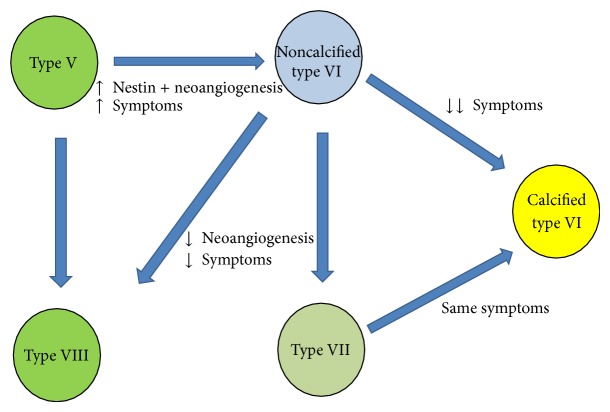
Flowchart illustrating the hypothetical plaque progression in relation to the Nestin-positive neoangiogenesis and calcification features. According to AHA classification [10], type V plaque is the uncomplicated fibroatheroma, type VI is the complicated plaque, type VII is the calcified plaque, and type VIII is the fibrotic plaque.

The reason why in the massively calcified plaques the neoangiogenesis and the histological complications do not affect symptomatology remains unclear, but it is possible that the hemorrhages and erosions found in these plaques might have a different pathogenesis. For example, it is possible that they can be due directly by the mechanical stresses of the calcified mass and not by immature neoangiogenesis and endothelial damage. Another possible explanation is that the “calcified type VI” plaques can represent an early stage of type VII plaques, in which the regressive process is more recent, and the complications have not disappeared yet (Figure 5).

The last result that emerged from our data is that 50% of the female patients had calcified plaques, showing a significantly lower incidence of symptoms and type VI plaques than the male patients. Ten years ago, Allison et al. have found 53% and 30% prevalence of “zero calcification” in female and male patients, respectively, before age of 50; after that age the prevalence of a diffuse vessel calcification increases, in a linear fashion in males and exponentially in females [16]. Actually, female sex hormones play an important role in bone tissue metabolism, increasing bone density and inhibiting osteoclast activity [20–22]. Nevertheless, it should be kept in mind that most women in our series were postmenopausal and their age at the moment of surgery did not differ from males (70.2 ± 9.5 versus 71.0 ± 8.5 years), so the question whether the postmenopausal hormone therapy might play a role in the pathophysiology of the atherosclerosis is still open.

*Study Limitations.* A limitation of our study is represented by heterogeneity in the sample size of each plaque group (e.g., 8 cases of type VII plaques were available). However, this is a monocentric perspective study, and our series reflects the incidence of the different plaque types in the general population. Moreover, the patients submitted to surgery generally have advanced plaques, and complicated type VI plaques are the most represented. Furthermore, there is a discrepancy between the age of the patients with plaques and the controls, due to obvious differences in the populations of multiorgan donors and atheromatous patients.

## 5. Conclusions

In conclusion, this study confirms that the Nestin+/WT1− phenotype characterizes the plaques with morphological features of instability, regardless of the actual amount of the neoangiogenesis (expressed as CD34-positive vessels). The plaques with massive calcifications show the same incidence of histological complications but with a lower incidence of neurological symptoms. Female patients show a much higher incidence of noncomplicated or calcified plaques, receiving* de facto* a sort of protection compared to male patients.

A possible indication emerging from these findings could be a comparison between the plaque dynamic imaging and the histological assessment of calcification, to evaluate the possibility of a presurgical risk stratification of patients, based on their sex, risk factors, and intraplaque calcification. The presurgical identification of those patients at major risk of developing stroke or brain lesions is likely to make the priority for endarterectomy more rational.

## Figures and Tables

**Table 1:** Technical characteristics of the antibodies used for immunohistochemistry.

Antibody	Clone	Manufacturer
Nestin	*10C2 (mouse IgG) *	Millipore
CD34	*QBEnd/10 (mouse IgG) *	Roche Ventana

**Table 2:** Summary of the baseline clinical characteristics of the 73 patients.

	Number of patients	Percentage
Stroke	11	15.1%
Transient ischemic attack	18	24.6%
Hypertension	65	89.0%
Dyslipidemia	51	69.9%
Diabetes	20	27.4%
Smoke	6	8.5%
Chronic ischemic cardiopathy	25	34.2%
Chronic obstructive pulmonary Disease	4	5.5%
Chronic renal failure	4	5.5%
Acetylsalicylic acid use	64	87.7%
Statins use	42	57.5%

**Table 3:** Densities of positivity of intraplaque neovessels for CD34 and Nestin in our series of plaques, sorted by calcification and by the occurrence of complications.

	Noncalcified plaques	Calcified plaques	Complicated plaques	Uncomplicated plaques
CD34 density	11.19 ± 3.84	7.52 ± 3.09	10.68 ± 3.79	8.99 ± 4.13
Mann-Whitney	*P* < 0.001		*P* = 0.111	

Nestin density	7.69 ± 3.77	4.59 ± 2.31	7.45 ± 3.71	5.31 ± 3.09
Mann-Whitney	*P* < 0.001		*P* = 0.015	
